# Chromosome differentiation patterns during cichlid fish evolution

**DOI:** 10.1186/1471-2156-11-50

**Published:** 2010-06-15

**Authors:** Andréia B Poletto, Irani A Ferreira, Diogo C Cabral-de-Mello, Rafael T Nakajima, Juliana Mazzuchelli, Heraldo B Ribeiro, Paulo C Venere, Mauro Nirchio, Thomas D Kocher, Cesar Martins

**Affiliations:** 1 UNESP - Universidade Estadual Paulista, Instituto de Biociências, Departamento de Morfologia, Botucatu, SP, Brazil; 2 UFMT - Universidade Federal de Mato Grosso, Instituto Universitário do Araguaia, Pontal do Araguaia, MT, Brazil; 3 Universidad de Oriente, Escuela de Ciencias Aplicadas del Mar, Boca de Rio, Venezuela; 4 University of Maryland, Department of Biology, College Park, MD 20742 USA

## Abstract

**Background:**

Cichlid fishes have been the subject of increasing scientific interest because of their rapid adaptive radiation which has led to an extensive ecological diversity and their enormous importance to tropical and subtropical aquaculture. To increase our understanding of chromosome evolution among cichlid species, karyotypes of one Asian, 22 African, and 30 South American cichlid species were investigated, and chromosomal data of the family was reviewed.

**Results:**

Although there is extensive variation in the karyotypes of cichlid fishes (from 2n = 32 to 2n = 60 chromosomes), the modal chromosome number for South American species was 2n = 48 and the modal number for the African ones was 2n = 44. The only Asian species analyzed, *Etroplus maculatus*, was observed to have 46 chromosomes. The presence of one or two macro B chromosomes was detected in two African species. The cytogenetic mapping of 18S ribosomal RNA (18S rRNA) gene revealed a variable number of clusters among species varying from two to six.

**Conclusions:**

The karyotype diversification of cichlids seems to have occurred through several chromosomal rearrangements involving fissions, fusions and inversions. It was possible to identify karyotype markers for the subfamilies Pseudocrenilabrinae (African) and Cichlinae (American). The karyotype analyses did not clarify the phylogenetic relationship among the Cichlinae tribes. On the other hand, the two major groups of Pseudocrenilabrinae (tilapiine and haplochromine) were clearly discriminated based on the characteristics of their karyotypes. The cytogenetic mapping of 18S ribosomal RNA (18S rRNA) gene did not follow the chromosome diversification in the family. The dynamic evolution of the repeated units of rRNA genes generates patterns of chromosomal distribution that do not help follows the phylogenetic relationships among taxa. The presence of B chromosomes in cichlids is of particular interest because they may not be represented in the reference genome sequences currently being obtained.

## Background

Teleost fishes have a successful history of diversification over the past 200 million years. The 23.000 species of teleosts make up almost half of all living vertebrates [1]. Perciformes represents the largest order of vertebrates with approximately 9.300 species. It includes more than 3.000 species of the family Cichlidae [1,2] that is one of the most species-rich families of vertebrates [3]. The natural distribution of cichlid fishes is centered on Africa, Latin America and Madagascar, with only a few species native to South India and the Middle East [4]. Mitochondrial genome sequences indicate that cichlids are closely related to the marine surfperches (Embiotocidae) and damselfishes (Pomacentridae), but not as previously thought, to wrasse and parrotfishes (Labridae and related families) [5]. Phylogenetic reconstructions are consistent with cichlid origins prior to Gondwanan landmass fragmentation 121-165 MYA, considerably earlier than the first known cichlid fossils from Eocene [5]. Cichlid fishes found in the lakes of Africa have served as model systems for the study of evolution [4,6,7]. Several species have received increasing scientific attention because of their great importance to tropical and subtropical aquaculture [8].

The family Cichlidae is a monophyletic group and the limits and interrelationships of all four subfamilies [Etroplinae (Indian and Madagascar), Ptychochrominae (Malagasy), Cichlinae (Neotropical region) and Pseudocrenilabrinae (African)] are well supported by molecular and morphological data [9]. The African (Pseudocrenilabrinae) and Neotropical (Cichlinae) cichlids are both monophyletic and represent sister groups [9]. The African Pseudocrenilabrinae cichlids are often assigned into pelmatochromine, haplochromine and tilapiine groups [10], but these groups are not recognized as valid taxonomic units. The Neotropical cichlids (Cichlinae) are monophyletic and are composed of 51 genera and 406 described species [11,12]. The most recently proposed phylogeny of the Cichlinae denotes the tribes Cichlini, Retroculini, Astronotini, Chaetobranchini, Geophagini, Cichlasomatini and Heroini [13].

The karyotypes of more than 135 species of cichlids have been determined. Although most species present a karyotype with 2n = 48, the diploid number ranges from 2n = 32 to 2n = 60 [14] (See Additional File 1: Available chromosomal data for the Cichlidae). African cichlids have a modal diploid number of 44 chromosomes whereas the Neotropical cichlids 2n = 48 chromosomes. Even though chromosomal data are known for several cichlid species, these data are not representative of the diversity of species in the group. Molecular cytogenetics approach to characterizing genome evolution has been applied to only a few species, principally *Oreochromis niloticus*. The aim of this work was to obtain chromosomal data for additional species of cichlids, including the mapping of 18S ribosomal RNA (rRNA) genes, and to compare these results to previous published chromosomal data and phylogenies, in order to correlate chromosomal rearrangements with particular phylogenetic transitions during the evolutionary history of the family Cichlidae.

## Results

### Basic cytogenetic analysis

#### Subfamily Etroplinae

**Figure 1:**
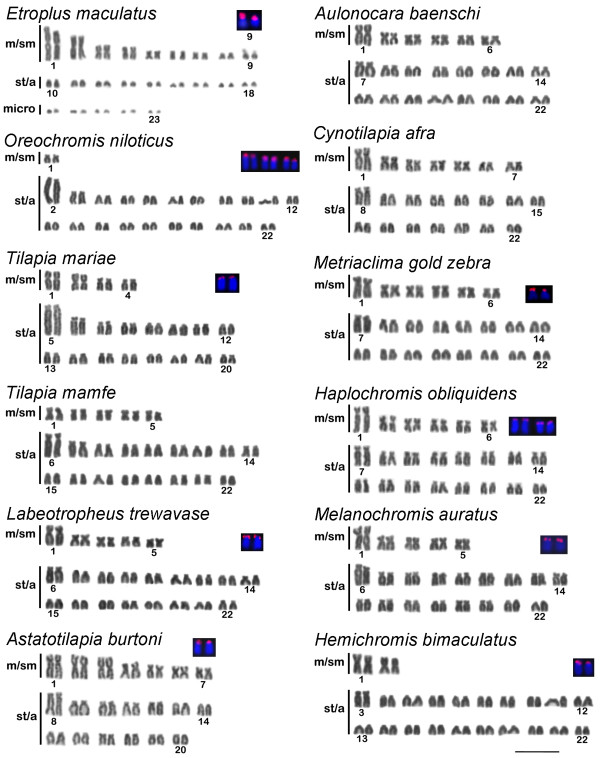
**Giemsa stained karyotypes of Asian and African cichlids and detail of the cytogenetic mapping of 18S rRNA genes**. The 18S rDNA probed chromosomes are shown, and the pair identified when it was possible. Scale bar, 10 μm.

The karyotype of *Etroplus maculatus *consists of 46 chromosomes including 18 m/sm (meta/submetacentric), 18 st/a (subtelo/acrocentric) and 10 microchromosomes (Table 1, Figure 1). The most remarkable characteristics of the *E. maculatus *karyotype were the presence of two outstanding large metacentric pairs, several small chromosomal pairs identified as m/sm or st/a, and five pairs of microchromosomes.

**Table 1:** Investigated African and South Asian Cichlids. n, number of analyzed animals; cr, chromosomes; st/a, subtelocentric/acrocentric; m/sm, meta/submetacentric; 1B, one B chromosome detected; 2B, two B chromosomes detected.

Subfamilies, Major groups and species	Origin	n	Karyotypic formulae	2n	rDNA sites
**Etroplinae**					
*Etroplus maculatus*	Petshop, Botucatu, SP, Brazil	03	18m/sm+18st/a+10micro	46	2cr, m/sm

**Pseudocrenilabrinae**					
Tilapiines					
*Oreochromis aureus*	Aquac. Facility, UMD, USA	03	2m/sm+42st/a	44	2cr, st/a
*Oreochromis mossambicus*	Aquac. Facility, UMD, USA	04	4m/sm+40st/a	44	3cr, st/a
*Oreochromis niloticus*	Tietê river, Botucatu, SP, Brazil CAUNESP, Jaboticabal, SP, Brazil; Aquac. Facility, UMD, USA	22	2m/sm+42st/a	44	6cr, st/a
*Oreochromis tanganicae*	Aquac. Facility, UMD, USA	01	2m/sm+42st/a	44	
*Tilapia mariae*	Aquac. Facility, UMD, USA	02	8m/sm+32st/a	40	2cr, st/a
*Tilapia mamfe*	Aquac. Facility, UMD, USA	01	10m/sm+34st/a	44	

Haplochromines					
*Astatotilapia burtoni*	Aquac. Facility, UMD, USA	03	14m/sm+26st/a	40	2cr, st/a
*Aulonocara baenschi*	Aquac. Facility, UMD, USA	03	12m/sm+32st/a	44	
*Cynotilapia afra*	Aquac. Facility, UMD, USA	01	14m/sm+30st/a	44	
*Gephyrochromis moorii*	Petshop, Botucatu, SP, Brazil	03	14m/sm+30st/a	44	
*Haplochromis livingstonii*	Petshop, Botucatu, SP, Brazil	01	14m/sm+30st/a	44	
*Haplochromis obliquidens*	Petshop, Botucatu, SP, Brazil	96	12m/sm+32st/a, 1B or 2B	44	4cr, st/a
*Labeotropheus trewavase*	Aquac. Facility, UMD, USA; Petshop, Botucatu, SP, Brazil	01	10m/sm+34st/a	44	2cr, st/a
*Melanochromis auratus*	Aquac. Facility, UMD, USA; Petshop, Botucatu, SP, Brazil	02	10m/sm+34st/a	44	2cr, st/a
*Metriaclima barlowi*	Aquac. Facility, UMD, USA	06	14m/sm+30st/a	44	2cr, st/a
*Metriaclima *gold *zebra*	Aquac. Facility, UMD, USA	04	12m/sm+32st/a	44	2cr, st/a
*Metriaclima lombardoi*	Aquac. Facility, UMD, USA; Petshop, Botucatu, SP, Brazil	22	14m/sm+30st/a, 1B	44	2cr, st/a
*Metriaclima pyrsonotus*	Aquac. Facility, UMD, USA	08	14m/sm+30st/a	44	
*Pseudotropheus tropheops*	Petshop, Botucatu, SP Brazil	01	14m/sm+30st/a	44	
*Pseudotropheus zebra*	Petshop, Botucatu, SP Brazil	01	14m/sm+30st/a	44	
*Pseudotropheus *sp	Petshop, Botucatu, SP Brazil	01	14m/sm+30st/a	44	

Hemichromines					
*Hemichromis bimaculatus*	Petshop, Botucatu, SP, Brazil	01	4m/sm+40st/a	44	2cr, st/a

#### Subfamily Pseudocrenilabrinae

In this work we sampled the tilapiines *Oreochromis aureus*, *O*. *mossambicus*, *O*. *niloticus*, *O*. *tanganicae, Tilapia mamfe *and *T*. *mariae *(Figure 1, Table 1). The karyotypes of the tilapiines investigated here are relatively conserved with 2n = 44 chromosomes for most species and the presence of a large st/a typical chromosome (pair 2 in *O. niloticus*, pair 5 in *T. mariae*, pair 6 in *T. mamfe*) (Figure 1). Reduction in the number of chromosomes was observed in *T. mariae *that shows 40 chromosomes with the presence of two atypical metacentric chromosome pairs (pairs 1 and 2) (Figure 1).

Most of the haplochromine species we analyzed had a karyotype composed of 2n = 44 (Figure 1, Table 1). *Astatotilapia burtoni *had a karyotype composed of 40 chromosomes, with 14 m/sm and 26 st/a chromosomes, and two large m/sm chromosome pairs (pairs 2 and 3) not observed in other haplochromines (Figure 1, Table 1). All the haplochromines have two typical large chromosome pairs, the first m/sm and the first st/a pairs.

When compared to the haplochromines and tilapiines, the karyotype of *Hemichromis bimaculatus *(Hemichromine) shows the same diploid number (2n = 44), but with only two m/sm chromosome pairs (Figure 1, Table 1). A large st/a (pair 3) and a large m/sm (pair 1) chromosome were also observed.

B chromosomes were detected in *Haplochromis obliquidens *and *Metriaclima lombardoi*. One or two large metacentric B chromosomes were present in 38 out of 96 analyzed specimens of *H. obliquidens *whereas one large B chromosome was detected in nine out of 22 animals sampled for *M. lombardoi *(Table 1).

#### Subfamily Cichlinae

**Figure 2:**
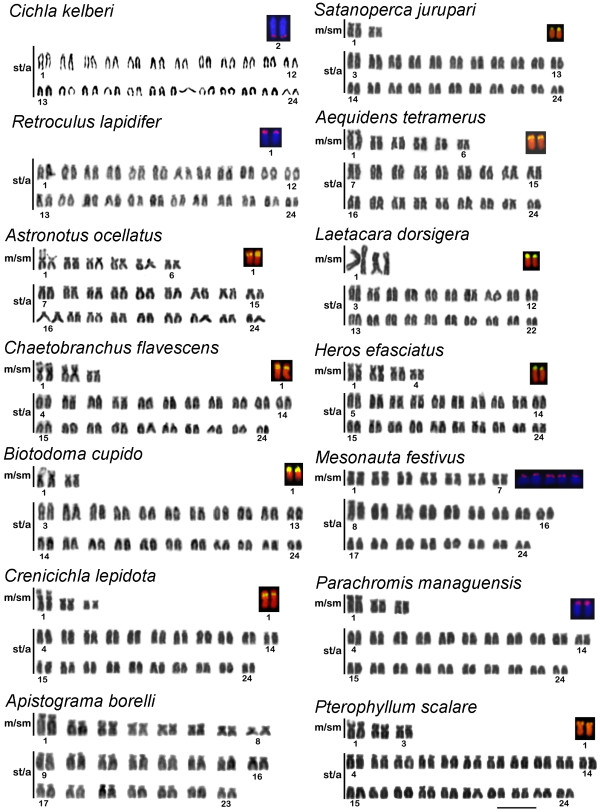
**Giemsa stained karyotypes of South American cichlids and detail of the cytogenetic mapping of 18S rRNA genes**. The 18S rDNA probed chromosomes are shown, and the pair identified when it was possible. Scale bar, 10 μm.

The karyotypes of *Cichla *species (Cichlini) and *Retroculus lapidifer *(Retroculini) presented 2n = 48 st/a chromosomes (Figure 2, Table 2). The karyotype of *Astronotus ocellatus *(Astronotinae) presents 12 m/sm chromosomes and *Chaetobranchus flavescens *(Chaetobranchini) shows 6 m/sm chromosomes (Figure 2, Table 2), both with 2n = 48.

**Table 2:** Investigated South American Cichlids (Cichlinae). n, number of analyzed animals; cr, chromosomes; st/a, subtelocentric/acrocentric; m/sm, meta/submetacentric.

Tribes and species	Origin	n	Karyotypic formulae	2n	rDNA sites
Cichlini					

*Cichla temensis*	Tocantins river, Tucuruí, TO, Brazil	17	48st/a	48	
*Cichla orinocensis*	Orinoco river, Caicara, Venezuela	01	48st/a	48	
*Cichla piquiti*	Araguaia river, São Felix do Araguaia, MT, Brazil	04	48st/a	48	
*Cichla kelberi*	Araguaia river, São Félix do Araguaia, MT, Brazil; Tietê river, Bariri, SP, Brazil	12	48st/a	48	2cr, st/a

Retroculini					
*Retroculus lapidifer*	Araguaia river, Barra do Garças, MT, Brazil	02	48st/a	48	2cr, st/a

Astronotini					
*Astronotus ocellatus*	Tietê river, Barra Bonita, SP, Brazil	09	12m/sm+36st/a	48	2cr, m/sm

Chaetobranchini					
*Chaetobranchus flavescens*	Araguaia river, São Félix do Araguaia, MT, Brazil	01	6m/sm+42st/a	48	2cr, m/sm

Geophagini					
*Apistogramma borellii*	Comprida lagoon, Aquidauana, MS, Brazil	05	16m/sm+30st/a	46	
*Biotodoma cupido*	Araguaia river, Barra do Garças, MT, Brazil Araguaia river, São Félix Araguaia, MT, Brazil	07	4m/sm+44st/a	48	2cr, m/sm
*Crenicichla lepidota*	Comprida Lagoon, Aquidauana, MS, Brazil	05	6m/sm+42st/a	48	2cr, m/sm
*Crenicichla strigata*	Araguaia river, Barra do Garças and São Félix do Araguaia, MT, Brazil	03	6m/sm+42st/a	48	
*Crenicichla britskii*	Olaria stream, Poloni, SP, Brazil	01	6m/sm+42st/a	48	
*Crenicichla *aff *britskii*	Olaria stream, Poloni, SP, Brazil	01	6m/sm+42st/a	48	
*Crenicichla *aff *haroldoi*	Olaria stream, Poloni, SP, Brazil	01	6m/sm+42st/a	48	
*Geophagus brasiliensis*	Olaria stream, Poloni, SP, Brazil Araquá stream, Botucatu, SP, Brazil Bonito river, Barra Bonita, SP, Brazil Paraitinguinha river, Salesópolis, SP, Brazil	07	2m/sm+46st/a	48	
*Geophagus proximus*	Araguaia river, Barra do Garças, MT, Brazil	04	4m/sm+44st/a	48	
*Geophagus *cf *proximus*	Tietê river, Buritama; Engenheiro Taveira river, Araçatuba (SP, Brazil)	04	4m/sm+44st/a	48	
*Geophagus surinamensis*	Orinoco river, Caicara, Venezuela	03	4m/sm+44st/a	48	
*Satanoperca jurupari*	Araguaia river, Barra do Garças, MT, Brazil Araguaia river, São Félix do Araguaia, MT, Brazil	16	4m/sm+44st/a	48	2cm, st/a

Cichlasomatini					
*Aequidens plagiozonatus*	Comprida lagoon, Aquidauana, MS, Brazil	09	12m/sm+36st/a	48	
*Aequidens tetramerus*	Araguaia river, Barra do Garças, MT, Brazil Araguaia river, São Félix do Araguaia, MT, Brazil	09	12m/sm+36st/a	48	2cr, st/a
*Cichlasoma facetum*	Campo Novo stream, Bauru; Paraitinguinha river, Salesópolis (SP, Brazil)	06	6m/sm+42st/a	48	
*Cichlasoma nigrofasciatum*	Petshop, Botucatu, SP, Brazil	13	8m/sm+40st/a	48	
*Cichlasoma paranaense*	Carrapato stream, Penápolis, SP, Brazil Batata stream, Miracatú, SP, Brazil Faú stream, Miracatú, SP, Brazil	08	6m/sm+42st/a	48	
*Laetacara dorsigera*	Bahia river, Pracinha, PR, Brazil	01	4m/sm+40st/a	44	2cr, st/a

Heroini					

*Heros efasciatus*	Araguaia river, Barra do Garças and São Félix Araguaia,(MT, Brazil)	03	8m/sm+40st/a	48	2cr, st/a
*Mesonauta festivus*	Araguaia river, Barra do Garças and São Félix Araguaia (MT, Brazil)	10	14m/sm+34st/a	48	6cr
*Parachromis managuensis*	Petshop, Botucatu, SP, Brazil	01	6m/sm+42st/a	48	2cr, m/sm
*Pterophyllum scalare*	Petshop, Botucatu, SP, Brazil	04	6m/sm+42st/a	48	2cr, m/sm
*Symphysodon aequifasciatus*	Petshop, Botucatu, SP, Brazil	02	46m/sm+4st/a+10micro	60	

The karyotypes of Geophagini species are similar to Chaetobranchini (Figure 2, Table 2). On the other hand, the karyotype of *Apistogramma borelli *presented a reduced number of chromosomes (2n = 46) and the presence of a higher number of m/sm chromosomes (eight pairs) compared to the non-geophagines (Figure 2).

Among the Cichlasomatini analyzed, *Laetacara dorsigera *showed a reduced chromosome number (2n = 44) with the presence of two outstanding large metacentric pairs (pairs 1 and 2) (Figure 2).

Heroini showed the broadest range of karyotype configurations. The cichlids *Heros efasciatus, Mesonauta festivus*, *Parachromis managuensis *and *Pterophyllum scalare *have 2n = 48 chromosomes with variations in the number of m/sm and st/a chromosomes (Figure 2, Table 2). The *Symphysodon aequifasciatus *karyotype is by far the most derived of all cichlids, with a highly increased number of chromosomes (2n = 60), including 10 microchromosomes.

### Cytogenetic mapping of 18S rRNA genes

The mapping of 18S rRNA genes was conducted in 26 representative Cichlidae species, including one Asiatic, 12 Africans and 13 South Americans (Figures 1 and 2, Tables 1 and 2). In the present work FISH proves identified the 18S rRNA gene in the terminal region of short arm of st/a chromosomes in almost all species. Exceptions were observed in *E. maculatus*, that presented this marker in the terminal region of a m/sm chromosome (pair 9) (Figure 1), and *C. kelberi *that harbours 18S rRNA genes in the terminal position of the long arm of the st/a chromosome pair 1 (Figure 2). In nine of the 12 African cichlids studied only two chromosomes were labeled by the 18S rRNA gene probe. Variation in the distribution of 18S sites were observed in three species: *O. niloticus *presented six labeled chromosomes (Figure 1); *O. mossambicus *showed three labeled chromosomes (data not shown); and *Haplochromis obliquidens *revealed four labeled chromosomes (Figure 1). For the South American species only one site of 18S rDNA was observed, except *M. festivus*, which presented five labeled chromosomes (Figure 2). Furthermore, the location of 18S sites in the short arms of a st/a chromosome pair appears to be common for *R. lapidifer*, *S. jurupari*, *A. tetramerus*, *L. dorsigera *and *H. efasciatus *(Figure 2). Another pattern for 18S rRNA gene position is represented by a large pair of m/sm with interstitial clusters in *A. ocellatus*, *Chaetobranchus flavescens *and *Crenicichla lepidota *(Figure 2). Terminal 18S rDNA sites were observed in *B. cupido*, *Parachromis managuensis *and *Pterophyllum scalare *(Figure 2). On the other hand, *Cichla kelberi*, has the 18S rDNA cluster located in the terminal position of the long arm of a large acrocentric pair (Figure 2), and *M. festivus *showed five chromosomes bearing 18S rDNA sites (Figure 2). Heteromorphic sites of the 18S rDNA was frequent in some species of the analyzed Neotropical cichlids as *A. ocellatus, C. flavescens, B. cupido, C. lepidota, S jurupari *and *H. efasciatus *(Figure 2). The 18S rDNA sites were mostly coincident with secondary constrictions observed in the Giemsa stained karyotypes (Figures 1, 2).

## Discussion

### Chromosome differentiation among cichlids

**Figure 3:**
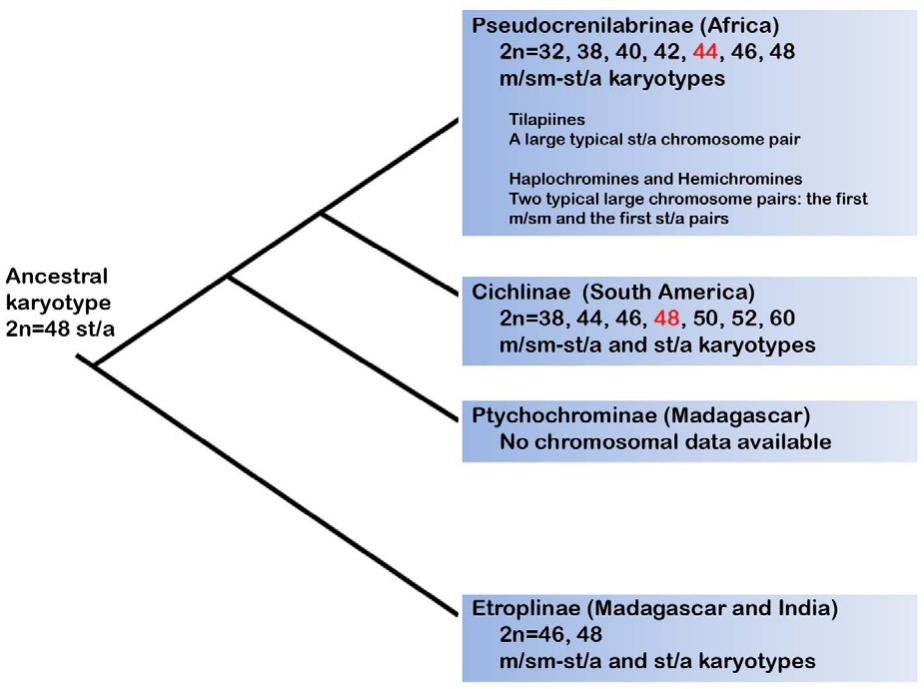
**Karyotype data plotted on the cladogram of the Cichlidae family**. The chromosome number variation is indicated and the modal chromosome numbers for the subfamilies are highlighted in red. The tree is based on the phylogeny proposed by [9].

The South American cichlids had distinct karyotypes compared to the Asian and African ones. The most remarkable characteristic is related to the modal chromosome number that is 48 for the South American and 44 for the African species [14] (See Additional File 1: Available chromosomal data for the Cichlidae clade). Besides that major pattern, small differences related to variations in the number of m/sm and st/a chromosomes are frequent and some species exhibit remarkable differences in their karyotypes related to the occurrence of specific chromosome rearrangements during their evolutionary history. Among the Pseudrocrenilabrinae clade, typical karyotype features discriminate the tilapiines from haplochromines and hemichromines (Figure 3).

The karyotypes of the Asian species *E. maculatus *and the South American *Symphysodon aequifasciatus *showed extensive chromosomal transformations when compared to the Perciformes basal karyotype. Etroplinae represents a sister clade of all other cichlids and *Symphysodon *(Heroini representative) represents a highly derived species inside the Cichlinae clade [13]. Another species of Etroplinae, *E. suratensis*, was described to contain 48 chromosomes [15], but the information provided is not clear concerning the morphology of the chromosomes. It seems, based on [15], that *E. suratensis *posses 48 st/a chromosomes, most similar to the basal karyotype of Perciformes [16]. The Etroplinae cichlids are quite morphologically distinct, exhibiting numerous specializations that are absent in all other cichlid lineages [15]. The subfamily Etroplinae was the first group to be isolated from the ancient cichlid group that was present in the Gondwanan supercontinent [9] and, the longest time of vicariance speciation could also account for the transformation to such a derived karyotype (Figure 3).

The African tilapiines present a highly conserved karyotype consisting of mostly st/a chromosomes and 2n = 44. Only a limited number of the known tilapia species have been karyotyped, but the species are closely related [17], and the existing evidence suggest that the tilapia karyotype is highly conserved [18,19]. To date, only four species have their karyotypes differing from 2n = 44, *T. mariae*, 2n = 40 (present paper), *T. sparrmanii*, 2n = 42 [20], *O. alcalicus*, 2n = 48 [21,22] and *O. karongae*, 2n = 38 [23]. The karyotype of *O. karongae *shows a reduced diploid number of chromosomes to 2n = 38 and differs from that found in most tilapia species. Different cytogenetic and genomic analysis previously conducted point to the reduction of chromosome number in *O. karongae *as a consequence of chromosome fusions involving three chromosome pairs in the ancestral of this species [23,24].

The presence of a large subtelocentric chromosome pair, that is the first st/a pair of the complement in *O. aureus*, *O. niloticus*, *T mariae*, *T. mamfe, O. mossambicus *and *O. tanganicae*, is an excellent marker for the group of tilapiines. The karyotpes of non-tilapiine species are recognizably distinct, despite having the same number of chromosomes. The large chromosome pair is the most remarkable characteristic of tilapiine karyotypes. Chromosome fusions are also believed to have occurred to create the largest chromosome pair of *O. niloticus *[25]. Chromosome fusions could also explain the reduction in the number of chromosomes in *T. mariae *to 40. The presence of two atypical metacentric chromosome pairs (pairs 1 and 2) in *T. mariae *suggests that these chromosomes originate from the fusion of small st/a chromosomes. These data support the hypothesis that chromosomal fusions occurred independently during the evolutionary history of tilapiines reducing the chromosome number from 2n = 44 as observed in several tilapia species.

Most of the haplochromine species we analyzed had a karyotype composed of 2n = 44. *Astatotilapia burtoni *had a karyotype composed of 40 chromosomes with the presence of two typical metacentric chromosome pair (2 and 3), which are probably the result of centric fusion of four small st/a chromosomes. These chromosomal rearrangements apparently occurred after the divergence of *Astatotilapia *from the other haplochromines that still retain 44 chromosomes in their karyotypes. Furthermore, the two largest pairs of chromosomes (first m/sm pair and first st/a pair) in the haplochromines stand out compared to the other chromosomes of the complement which make them good markers for this group. The chromosome information of haplochromines is in agreement with their phylogenetic divergence of other African Pseudocrenilabrinae [26].

The Neotropical cichlids have a modal number of 2n = 48, with the exception of *Apistograma borelli *(2n = 46), *Laetacara dorsigera *(2n = 44), and *Symphysodon aequifasciatus *(2n = 60). The chromosomal number for South American cichlids ranges from 2n = 48 st/a chromosomes in *Cichla *spp., considered the most basal karyotype, to 2n = 60 (46m/sm, 4st/a and 10 microchromosomes) in *Symphysodon aequifasciatus*. *Cichla *(Cichlini) and *Retroculus *(Retroculini) presented a karyotype structure composed only of st/a, similar to the proposed Perciformes ancestral karyotype [16]. In the most recent proposed phylogeny for South American cichlids, a clade composed of Cichlini and Retroculini was recovered as the sister group to all other South American cichlids [13]. On the other hand, *Symphysodon *exhibited the most derived karyotype condition compared to the proposed Perciformes ancestral karyotype and also occupy a derived position in the phylogeny of the group [13].

The karyotype formula 2n = 48 st/a elements is characteristic of Perciformes, as observed in Sciaenidae [27,28], Pomacentridae [29] and Haemulidae [30]. These data suggest that *Cichla *and *Retroculus *retain the ancestral karyotype pattern of the group (2n = 48 st/a). The ancestral karyotype has undergone major changes in its macro-structure in some lineages, which has led to the extensive karyotype diversification that is currently observed among cichlids. This observation is consistent with several proposed phylogenies for the family [[13], for review], which generally include *Cichla *and *Retroculus *as sister group of the other Neotropical cichlids.

The derived chromosomal patterns of *Symphysodon *probably results from rearrangement involving chromosomal pericentric inversions, translocations and fissions/fusions [31,32]. Repetitive DNA elements seem to have contributed to the chromosomal diversification of *Symphysodon *karyotypes in relation to other cichlids [33].

Pericentric inversions are thought to be the main mechanism contributing to changes in the basal chromosome arm size of Perciformes [34,35]. Other mechanisms of chromosomal rearrangement and translocation probably have contributed to the karyotypic diversification of South American cichlids. The chromosome number variation observed in some species suggests that events of chromosomal translocation followed by chromosome fission and fusion were also involved. It remains to be investigated whether specific events of chromosome rearrangements (fusion, fission, inversion) that occurred during the evolutionary history of cichlids are related to particular characteristics of their genomes.

### B chromosome in African cichlids

In addition to the standard cichlid karyotype pattern, large metacentric B chromosomes were observed at high frequency among specimens of *H. obliquidens *and *M. lombardoi*. One notable characteristic of the B chromosomes found in these species is their large size, which is almost the same as the largest pair of the A complement. Information concerning the occurrence and the genomic content of B chromosomes among African cichlids has just recently been reported for *H. obliquidens *[36]. The occurrence of supernumerary chromosomes has been described for species of diverse fish groups. In general the supernumerary chromosomes of fishes vary in number and morphology. Among cichlids, supernumerary chromosomes have been described in only a few species from South America. They were first described for male germinative cells of *Gymnogeophagus balzanii *[37] and for species of *Geophagus brasiliensis*, *Cichlasoma paranaensis *and *Crenicichla niederleinii *[38]. Small supernumerary chromosomes were also described for *Cichla monoculus*, *Cichla *sp. and *Crenicichla reticulata *[[39], for review].

Since some African cichlid species genomes are being completely sequenced [40], it will be of particular interest to investigate the occurrence of B chromosomes among cichlid species for future genomic analyses.

### Cytogenetic mapping of 18S rRNA genes

The ancestral condition for the location of the nuclear organizer region (NOR) in cichlids, is supposed to be one pair of chromosomes [[14], for review] (See Additional File 1: Available chromosomal data for the Cichlidae clade). But these results were obtained mostly by silver nitrate staining, that might not correspond to the real genomic organization for the 18S rRNA genes. In the present work FISH probing with the 18S rRNA gene showed that the Asian cichlid *E. maculatus*, despite its rearranged karyotype, has the ancestral condition of 18S rRNA gene cluster localized in just one pair of chromosomes. In African cichlids it seems that different rearrangements involving the 18S rDNA bearing chromosome pair have occurred. Compared to the ancestral hypothetical condition, *O. niloticus *exhibit the most derived condition of the African species, with multiple sites of 18S rRNA genes spread in the short arms of 6 st/a chromosomes, whereas the other African species evidenced a lower number of sites similar to the proposed ancestral condition.

With the exception of *M. festivus*, which has five marked chromosomes, the 18S rRNA gene probing in all Neotropical cichlids revealed a single pair of 18S rDNA bearer chromosomes, which is probably the ancestral condition for the group [37]. The 18S rRNA genes were previously mapped in one chromosome pair in *G. brasiliensis *and *C. facetum *[41]. Furthermore, the location of 18S rRNA gene clusters in the short arms of a st/a chromosome pair appears to be common for several species (*A. tetramerus, L. dorsigera *and *H. efasciatus*). Another pattern for 18S rRNA gene position is represented by a large pair of m/sm with interstitial clusters, probably produced by paracentric inversion, in *A. ocellatus*, *C. flavescens, B. cupido, C. lepidota*. On the other hand, despite having the supposed ancestral karyotype, *Cichla kelberi *has the 18S rDNA cluster located in the terminal position of the long arm of a large acrocentric pair, what seems to be a derived condition for the group. Previous data on 18S rDNA distribution on species of *Symphysodon *(*Symphysodon aequifasciatus*, *Symphysodon discus *and *Symphysodon haraldi*) showed variations from 2-5 sites [42]. Considering that *Symphysodon *(Heroini representative) represents the most derived taxa inside the Cichlinae [13], the spread of rDNA sites seems to have followed the diversification of the subfamily.

The 18S rDNA sites were mostly coincident with secondary constrictions observed in the Giemsa stained karyotypes. Heteromorphic sites of the 18S rDNA was frequent in some species of the analyzed Neotropical cichlids as *A. ocellatus, C. flavescens, B. cupido, C. lepidota, S jurupari *and *H. efasciatus*, that could indicate a process of unequal crossover or differential rDNA amplification between the homologous chromosomes.

The variation observed in the chromosomal distribution of rDNA sites is not informative in relation to the phylogeny of the family Cichlidae. Repeated DNAs like the major ribosomal RNA multigene families are subject to the action of several molecular mechanisms and are thought to be the most rapidly evolving components of eukaryotic genomes [43,44]. The highly dynamic evolutionary rate of repeated elements generated patterns of chromosomal organization that do not represent the phylogeny of the groups. Among fishes, the chromosomal distribution of rDNA clusters can be informative for comparative analysis of closely related species or even to the characterization of populational variations. The distribution of 18S rDNA in three species of *Symphysodon *(*Symphysodon aequifasciatus*, *Symphysodon discus *and *Symphysodon haraldi*), showed intra and interspecies variations both in the number and in the size of the sites [42]. So, care should be exercised in making phylogenetic inferences based on rDNA cytogenetic map data, at least for comparisons involving higher taxa.

## Conclusions

Although different events of chromosomal rearrangements have acted during the evolutionary history of cichlids, it was possible to identify characteristic chromosome patterns for the subfamilies Pseudocrenilabrinae (African) and Cichlinae (American). The karyotype analyses did not clarify the phylogenetic relationship among the Cichlinae tribes. On the other hand, the two major groups of the African Pseudocrenilabrinae, tilapiine and haplochromine, were clearly discriminated based on the characteristics of their karyotype. The cytogenetic mapping of 18S rRNA genes did not identify markers useful for studying the chromosomal diversification of the Cichlidae clade, possible as a consequence of the rapid evolution of the repeated units of rRNA genes that generates divergent patterns of chromosomal distribution even among closely related species.

## Methods

### Specimens and chromosome preparation

In the present work we analyzed one species of South Indian cichlid obtained from a commercial source, 22 species of African cichlids, some obtained from commercial sources and some from wild stocks (mainly from the Lake Malawi, East Africa) maintained at the Tropical Aquaculture Facility of University of Maryland, USA (Table 1), and 30 South American cichlid species collected from several South American hydrographic systems (Table 2). The fishes were euthanized with a lethal dose of benzocaine followed by spinal section (Protocol 01204 - Committee of Ethical in Animal Experimentation - UNESP - São Paulo State University, Brazil) before removal of kidneys for chromosome preparation.

Mitotic chromosome preparations were obtained from kidney according to [45]. In attempt to obtain a larger number of metaphases of good quality, animals were injected with a bread yeast solution 12-24 hours prior the dissection. Animals were treated with a 0.025% solution of colchicine (1 ml/100 g weigh body) 40 minutes before euthanasia and chromosome preparation. The kidney tissues were dissected and the cells dissociated with the use of a syringe in a hypotonic solution of KCl 0.075% and kept in this solution for 30 to 50 min. The cells were fixed in 3:1 methanol-acetic acid and used to prepare slides that were stained with Giemsa solution 5% in phosphate buffer at pH 7 for 10 min.

### Fluorescence *in situ *hybridization

The 18S rRNA gene was isolated via PCR (Polymerase Chain Reaction) from the genome of *O. niloticus *and used as probes for fluorescence *in situ *chromosome hybridization (FISH) in 26 representative species of Cichlidae including Asian, African and South American species (Tables 1 and 2). Copies of the 18S rRNA gene were amplified with the primers 18Sf (5'-CCG CTT TGG TGA CTC TTG AT) and 18Sr (5'-CCG AGG ACC TCA CTA AAC CA), which were designed based upon the sequence of the catfish *Ictalurus punctatus *(GenBank accession number AF021880) to amplify an approximately 1,400 base pairs (bp) DNA segment of the 18S rRNA gene.

Mitotic chromosomes were subjected to FISH [46] (Pinkel et al. 1986) using the PCR products from the 18S rRNA genes as probes. The probes were labeled by nick translation with biotin-14-dATP (Invitrogen) or digoxigenin-11-dUTP (Roche). The metaphase chromosome slides were incubated with RNase (40 μg/ml) for 1.5 h at 37°C. After, the chromosomal DNA was denatured in 70% formamide, 2× SSC for 4 min at 70°C. The hybridization mixtures, which contained 100 ng of the denatured probe, 10 mg/ml dextran sulfate, 2× SSC and 50% formamide in a final volume of 30 μl, were dropped on the slides, and the hybridization was performed overnight at 37°C in a 2× SSC moist chamber. Post-hybridization washes were carried out at 37°C in 2× SSC, 50% formamide for 15 min, followed by a second wash in 2× SSC for 15 min and a final wash at room temperature in 4× SSC for 15 min. Detection of the labeled probes was carried out with Avidin-FITC (Sigma) or antidigoxigenin-rhodamin (Roche). Chromosomes were counterstained with propidium iodide (0.2%) or DAPI (Sigma) diluted in antifade (Vector).

### Chromosome analysis

The chromosome spreads were analyzed using an Olympus BX 61 microscope, and the images were captured with the Olympus DP71 digital camera with the software Image-Pro MC 6.0. Karyotypes were arranged in order of decreasing chromosome size and the chromosomes classified as meta/submetacentric (m/sm), subtelo/acrocentrics (st/a) and microchromosomes.

## Authors' contributions

PCV and MN carried out the field work and contributed in the chromosome preparations. ABP, IAF and HBR carried out the chromosome analysis and the karyotype organization. DCCM, RTN and JM carried out the molecular cytogenetic analysis. ABP, DCCM and JM helped to draft the manuscript. TDK participated in the design of the study, chromosome preparations and preparation of the manuscript. CM conceived of the study, and participated in its design and coordination, drafted and revised the manuscript. All authors read and approved the final manuscript.

## Supplementary Material

Additional file 1 **Available chromosomal data for the Cichlidae clade**. n, haploid number; 2n, diploid number; KF, karyotypic formulae; NOR, nucleolus organizer region; m/sm, meta-submetacentric chromosomes; st/a, subtelo-acrocentric chromosomes; a, acrocentric chromosomes. The species are distributed in subfamilies and tribes according to [13]. The data presented here were updated from [14]. Click here for file
