# Supplementary material for: Chromosome differentiation patterns during cichlid fish evolution
Source: BMC Genet. 2010 Jun 15;11:50. doi: 10.1186/1471-2156-11-50 (PMC2896337; doi:10.1186/1471-2156-11-50)
Supplement: Additional file 1 — Available chromosomal data for the Cichlidae clade. n, haploid number; 2n, diploid number; KF, karyotypic formulae; NOR, nucleolus organizer region; m/sm, meta-submetacentric chromosomes; st/a, subtelo-acrocentric chromosomes; a, acrocentric chromosomes. The species are distributed in subfamilies and tribes according to [13]. The data presented here were updated from [14]. [file 1471-2156-11-50-S1.PDF]

Additional File 1: Available chromosomal data for the Cichlidae clade. n, haploid number; 2n, diploid number; KF, karyotypic formulae; NOR, nucleolus organizer region; m/sm, meta-submetacentric chromosomes; st/a, subtelo-acrocentric chromosomes, a, acrocentric chromosomes. The species are distributed in subfamilies and tribes according to [13]. The data presented here were updated from [14].

Subfamilies, Tribes and Species	Origin	n	2n	KF	NOR sites	References
**Etroplinae**						
Etroplus maculatus			46			[15]
Etroplus maculatus	Commercial source, Brazil		46	18m/sm+18st/a+ 10micro	2	present paper
Etroplus suratensis			48	48 st/a**		[15]
**Pseudocrenilabrinae**						
Astatotilapia burtoni	Aquac. Facility, UMD, (MD, USA)		40	14m/sm+26st/a	2	present paper
Aulonocara baenschi	Aquac. Facility, UMD, (MD, USA)		44	12m/sm+32st/a		present paper
Cynotilapia afra	Aquac. Facility, UMD, (MD, USA)		44	14m/sm+30st/a		present paper
Gephyrochromis moorii	Commercial source, Brazil		44	14m/sm+30st/a		present paper
Haplochromis burtoni	Africa		40	14m/sm+26st/a		[20]
Haplochromis flaviijosephi	Sea of Galilee		44	10m/sm+34st/a		[47]
Haplochromis livingstonii	Commercial source, Brazil		44	14m/sm+30st/a		present paper
Haplochromis obliquidens	Commercial source, Brazil		44	12m/sm+32st/a*	4	present paper
Hemichromis bimaculatus			44			reviewed in [48,49]
Hemichromis bimaculatus	Commercial source, Brazil		44	4m/sm+40st/a	2	present paper
Labeotropheus trewavase	Aquac. Facility, UMD, (MD, USA)		44	14m/sm+30st/a	2	present paper
Melanochromis auratus	Africa		46	12m/sm+34st/a		[20]
Melanochromis auratus	Aquac. Facility, UMD, (MD, USA)		44	10m/sm+34st/a	2	present paper
Metriaclima barlowi	Aquac. Facility, UMD, (MD, USA)		44	14m/sm+30st/a	2	present paper
Metriaclima gold zebra	Aquac. Facility, UMD, (MD, USA)		44	12m/sm+32st/a	2	present paper
Metriaclima lombardoi	Aquac. Facility, UMD, (MD, USA)		44	14m/sm+30st/a*	2	present paper
Metriacilma pyrsonotus	Aquac. Facility, UMD, (MD, USA)		44	14m/sm+30st/a		present paper
Neolamprologus leleupi			48			reviewed in [48]
Oreochromis karongae	Stirling University (Scotland)		38			[23]
Oreochromis macrochir			44			reviewed in [49]
Oreochromis macrochir	Zaire		44	6m/sm+38st/a		[50]
Oreochromis alcalicus			48			reviewed in [49]
Oreochromis andersonii			44	4m/sm+40st/a		[50]
Oreochromis andersoni			44	6m/sm+38st/a		[50]
Oreochromis aureus	Sea of Galilee		44	10m/sm+34st/a		[47]
Oreochromis aureus			44	6m/sm+38st/a		[20]
Oreochromis aureus	Aquac. Facility, UMD, (MD, USA)		44	2m/sm+42st/a	2	present paper
Oreochromis mossambicus			44	44m/sm		[49]
Oreochromis mossambicus	Aquac. Facility, UMD, (MD, USA)		44	4m/sm+40st/a	3	present paper
Oreochromis mossambicus			44			[51, 52]
Oreochromis niloticus		22	44			[17, 49, 53, 54]
Oreochromis niloticus			40			[55]
Oreochromis niloticus	Tietê river, Botucatu; CAUNESP, Jaboticabal (SP, Brazil); Aquaculture Facility, UMD (MD, USA)		44	2m/sm+42st/a	6	present paper
Oreochromis tanganicae	Aquac. Facility, UMD, (MD, USA)		44	2m/sm+42st/a		present paper
Pelvicachromis pulcher			48			reviewed in [49]
Pseudocrenilabrus multicolor			44			reviewed in [49]
Pseudotropheus tropheops	Commercial source, Brazil		44	14m/sm+30st/a	2	present paper
Pseudotropheus zebra	Commercial source, Brazil		44	14m/sm+30st/a		present paper
Pseudotropheus sp.	Commercial source, Brazil		44	14m/sm+30st/a		present paper
Sarotherodon galilaeus			44			[49, 55, 56]
Sarotherodon galilaeus			44	6m/sm+38st/a		[50]
Sarotherodon mossambica	África		44	6m/sm+38st/a		[20]
Sarotherodon multifasciatus			44			[57]
Tilapia busumana			44			[57]
Tilapia congica	Zaire		44	10m/sm+34st/a		[50]
Tilapia guineensis			44	8m/sm+36st/a		[50]
Tilapia macrocephala			32			[58]
Tilapia mariae			40	4m/sm+36st/a		[50]
Tilapia mariae	Africa		40	8m/sm+32st/a		[20]
Tilapia mariae	Aquac. Facility, UMD, (MD, USA)		40	8m/sm+32st/a		present paper
Tilapia mamfe	Aquac. Facility, UMD, (MD, USA)		44	10m/sm+34st/a	2	present paper

Tilapia rendalli	Ribeirão Preto (SP, Brazil)		44	16m/sm+28st/a		[59]
Tilapia sparrmanii	Zaire		42	8m/sm+34st/a		[50]
Tilapia sparrmanii	Africa		42	8m/sm+34st/a		[20]
Tilapia zillii			38			[56]
Tilapia zillii	Sea of Galilee		44	10m/sm+34st/a		[49]
Tristramella sacra	Sea of Galilee		44	6m/sm+38st/a		[49]
Tristramella simonis	Sea of Galilee		44	6m/sm+38st/a		[49]
**Cichlinae**						
Cichla sp.	Uatumã River (AM, Brazil)		48	48a*	2	[39, 60]
Cichla kelberi	São Félix do Araguaia, MT; Bariri, SP (Brazil)		48	48st/a	2	present paper
Cichla monoculus	Uatumã river (AM, Brazil)		48	48a*	2	[39, 60]
Cichla temensis	Commercial source		48	48st/a	2	[61]
Cichla temensis	Uatumã river (AM, Brazil)		48	48a	2	[60]
Cichla temensis	Tocantins river, Tucuruí reservoir, (TO, Brazil)		48	48st/a	2	present paper
Cichla orinocensis	Orinoco river (Venezuela)		48	48st/a	2	present paper
Cichla piquiti	Araguaia river, São Felix do Araguaia (MT, Brazil)		48	48st/a	2	present paper
**Retroculini**						
Retroculus lapidifer	Araguaia river, Barra do Garças (MT, Brazil)		48	6m/sm+42st/a	2	present paper
**Astronotinae**						
Astronotus ocellatus			48		2	reviewed in [49]
Astronotus ocellatus	Commercial source	24	48	6m/sm+42st/a		[61, 62]
Astronotus ocellatus	Miranda (MS, Brazil)	24	48	12m/sm+36st/a		[63, 64]
Astronotus ocellatus	Manaus (AM, Brazil)		48	12m/sm+36st/a	2	[63, 64]
Astronotus ocellatus	Tietê river, Barra Bonita (SP, Brazil)		48	12m/sm+36st/a		present paper
Chaetobranchopsis australis	Miranda river (MS, Brazil)		48	48st/a	2	[63, 64]
Chaetobranchus flavescens	Araguaia river, São Félix do Araguaia (MT, Brazil)		48	6m/sm+42st/a		present paper
**Geophaginae**						
Acarichthys heckelii	Commercial source		48	6m/sm+42st/t	1	[61]
Apistogramma agassizii	Commercial source	20	46	24m/sm+22st/a		[61]
Apistogramma agassizii		23				[62]
Apistogramma borellii	Commercial source	23	38	22m/sm+16st/a		[61]
Apistogramma borellii		23				[62]
Apistogramma borellii	Comprida lagoon, Aquidauana (MS, Brazil)		46	16m/sm+30st/a		present paper
Apistogramma cacatuoides						[62]
Apistogramma ortmanni	Commercial source	19	46	24m/sm+22st/a		[61]
Apistogramma ortmanni		24				[62]
Apistogramma pertensis						reviewed in [49]
Apistogramma steindachneri			46			reviewed in [49]
Biotodoma cupido	Araguaia river, Barra do Garças and São Félix Araguaia (MT, Brazil)		48	4m/sm+44st/a	2	present paper
Crenicichla britskii	Jupiá river (PR, Brazil)		48	8m, sm+40st/a	2	[65]
Crenicichla britskii	Olaria stream, Poloni (SP, Brazil)		48	6m/sm+42st/a		present paper
Crenicichla aff *britskii*	Olaria stream, Poloni, (SP, Brazil)		48	6m/sm+42st/a		present paper
Crenicichla cincta	Catalão lake (AM, Brazil)		48	8m/sm+40st/a	2	[65]
Crenicichla cf *johanna*	Catalão lake (AM, Brazil)		48	8m/sm+40st/a	2	[65]
Crenicichla inpa	Amazon region Brazil)		48	6m/sm+42st/a	2	[65]
Crenicichla aff *haroldoi*	Olaria stream, Poloni (SP, Brazil)		48	6m/sm+42st/a		present paper
Crenicichla lacustris	Registro (SP, Brazil)	24	48	6m/sm+42st/a	2	[63, 64]
Crenicichla lepidota		24				[62]
Crenicichla lepidota	Miranda river (MS, Brazil)		48	6m/sm+42st/a	2	[63, 64]
Crenicichla lepidota	Commercial Source		48	6m/sm+42st,t		[61]
Crenicichla lepidota	Paraná river (PR, Brazil)		48	6m/sm+42st/a	4	[38]
Crenicichla lepidota	Misiones (Argentina)		48	6m/sm+42st/a	2	[66]
Crenicichla lepidota	Comprida lagoon, Aquidauana (MS, Brazil)		48	6m/sm+42st/a	2	present paper
Crenicichla lucius	Commercial source		48			[61]
Crenicichla lugubris	Catalão lake (AM, Brazil)		48	8m/sm+40st/a	2	[65]
Crenicichla niederleinii	Paraná river (PR, Brazil)		48	14m/sm+34st/a*	2	[38]
Crenicichla niederleinii	Tibagí river (PR, Brazil)		48	10m/sm+40st/a	2	[68]
Crenicichla niederleinii	Misiones (Argentina)		48	6m/sm+42st/a	2	[66]
Crenicichla notophthalmus	Commercial source		48	6m/sm+42st/a		[61]
Crenicichla reticulata	Careiro (AM, Brazil)		48	6m/sm+42st/a	2	[65]
Crenicichla reticulata	Uatumã river (AM, Brazil)		48	6m/sm+42st/a*	2	[39]
Crenicichla semifasciata	Miranda (MS, Brazil)	24	48	6m/sm+42st/a	2	[63, 64]
Crenicichla semifasciata	Misiones (Argentina)		48	6m/sm+42st/a		[66]
Crenicichla sexatilis	Uruguai	24	48	4m/sm+44st/a		[67]
Crenicichla sp.	São Benedito river, Itajaí (SC, Brazil)		48	8m/sm+40 st/a	2	[68]
Crenicichla strigata	Commercial source	24	48	6m/sm+42st/a		[61]
Crenicichla strigata	Barra do Garças and São Félix do Araguaia (MT, Brazil)		48	6m/sm+42st/a		present paper

Crenicichla vittata	Miranda (MS, Brazil)		48	6m/sm+42st/a	2	[63, 64]
Dicrossus filamentosus	Commercial source		46	12m/sm+34st/a		[61]
Dicrossus maculatus		23				[62]
Geophagus brasiliensis	Ribeirão Preto (SP, Brazil)		48	3m/sm+45st/a		[59]
Geophagus brasiliensis			48			reviewed in [49]
Geophagus brasiliensis	Brotas (SP, Brazil)	24	48	2m/sm+46st/a	2	[63, 64]
Geophagus brasiliensis	São Carlos (SP, Brazil)	24	48	2m/sm+46st/a	2	[63, 64]
Geophagus brasiliensis	Pirassununga (SP, Brazil)	24	48	2m/sm+46st/a	2	[63, 64]
Geophagus brasiliensis	Registro (SP, Brazil)	24	48	2m/sm+46st/a	2	[63, 64]
Geophagus brasiliensis	Commercial source		48	4m/sm+44st/a		[61]
Geophagus brasiliensis	Paranapanema river (Brazil)		48	8m/sm+40st/a*	2	[38]
Geophagus brasiliensis	Paranapanema river, Ribeira river, Tibagi river (Brazil)		48	3sm+21st/a	2	[41]
Geophagus brasiliensis	Tibagi river (PR, Brazil)		48	2sm+22st/a	1	[69, 70]
Geophagus brasiliensis	Saco da Alemoa and Gasômetro (RS, Brazil)		48	4sm+44st/a	2	[69, 70]
Geophagus brasiliensis	Olaria stream, Poloni; Araquá stream, Botucatu; Bonito river, Barra Bonita; Paraitinguinha river, Salesópolis (SP, Brazil.		48	2m/sm+46st/a		present paper
Geophagus proximus	Araguaia river, Barra do Garças (MT, Brazil)		48	4m/sm+44st/a		present paper
Geophagus cf *proximus*	Tietê river, Buritama; Engenheiro Taveira river, Araçatuba (SP, Brazil)		48	4m/sm+44st/a		present paper
Geophagus surinamensis	Amazon region (AM, Brazil)	24	48	4m/sm+44st/a	2	[63, 64]
Geophagus surinamensis	Commercial source		48	4m/sm+44st/a		[61]
Geophagus surinamensis	Orinoco river (Venezuela)		48	4m/sm+44st/a		present paper
Gymnogeophagus gymnogenys	Saco da Alemoa and Barra do Ribeiro (RS, Brazil)		48	4m+44st-a	4	[69, 70]
Gymnogeophagus gymnogenys	Gasômetro (RS, Brazil)		48	6m+42st-a	2	[69, 70]
Gymnogeophagus balzanii	Miranda (MS, Brazil)	24	48	2m/sm+46st/a*	2	[37, 63, 64]
Gymnogeophagus labiatus	Saco da Alemoa and Forqueta river (RS, Brazil)		48	4m+4sm+40st/a	2	[69]
Mikrogeophagus ramirezi		24				reviewed in [49], [62]
Satanoperca jurupari	Commercial source		48	4m/sm+44st/a		[61]
Satanoperca jurupari	Araguaia river, Barra do Garças and São Félix do Araguaia (MT, Brazi)l		48	4m/sm+44st/a	2	present paper
Satanoperca pappaterra	Paraná river (Brazil)		48	6m/sm+42st/a	2	[38]
**Cichlasomatinae**						
Aequidens metae			48	6m/sm+42st/a		[61]
Aequidens plagiozonatus	Comprida lagoon, Aquidauana (MS, Brazil)		48	12m/sm+36st/a		present paper
Aequidens pulcher		24				[62]
Aequidens tetramerus	Araguaia river, Barra do Garças and São Félix do Araguaia (MT, Brazil)		48	12m/sm+36st/a	2	present paper
Amphilophus citrinellus		24				[62]
Amphilophus citrinellus			48	36m/sm+12st/a		[71]
Amphilophus citrinellus			48			reviewed in [49]
Amphilophus citrinellus			48	8m/sm+40st/a		[61]
Amphilophus macracanthus		24				[62, 73]
Amphilophus macracanthus			48			reviewed in [49]
Amphilophus macracanthus			48	6m/sm+42st/a		[61]
Archocentrus centrarchus			48	6m/sm+42st/a		[61]
Archocentrus nigrofasciatus		24				[62]
Archocentrus nigrofasciatus			48			reviewed in [49]
Archocentrus nigrofasciatus	Cuarto river, Costa Rica		48	4m/sm+44st/a		[61]
Archocentrus septemfasciatus	Cuarto river, Costa Rica		48	6m/sm+42st/a		[61]
Archocentrus spilurus		24				[62]
Bujurquina vittata			44	26m/sm+18st/a		[61]
Caquetaia kraussii			50	6m/sm+44st/a		[61]
Cichlasoma beani	Mexico		48	6m/sm+42st/a		[61]
Cichlasoma bimaculatum			48	6m/sm+42st/a		[61]
Cichlasoma facetum	Uruguay	24	48	8m/sm+40st/a		[67]
Cichlasoma facetum	Registro (SP, Brazil)	24	48	10m/sm+38st/a	2	[63, 64]
Cichlasoma facetum	Rio Claro (SP, Brazil)	24	48	10m/sm+38st/a	2	[63, 64]
Cichlasoma facetum	Tarumã lake, Ponta Grossa (PR, Brazil)		48	5sm+19st/a	1	[41]
Cichlasoma facetum	Campo Novo stream, Bauru; Paraitinguinha river, Salesópolis (SP, Brazil)		48	6m/sm+42st/a		present paper
Cichlasoma nigrofasciatum	Commercial Source, Brazil		48	8m/sm+40st/a		present paper
Cichlasoma octofasciatus			48			reviewed in [49]
Cichlasoma octofasciatus	Londrina (PR, Brazil)		48	6m/sm+42st/a		[61]
Cichlasoma paranaense			48	20m/sm+28st/a*	2	[38]

Cichlasoma paranaense	Carrapato stream, Penápolis; Batata stream, Miracatú; Faú stream, Miracatú (SP, Brazil)		48	6m/sm+42st/a		present paper
Cichlasoma portalegrense			24			[62]
Cichlasoma salvini	Belize		52			reviewed in [49]
Cichlasoma salvini			52	28m/sm+24st/a		[61]
Cichlasoma sp. A	Paraguai river (MT, Brazil)	24				[62]
Cichlasoma trimaculatus			48	6m/sm+42st/a		[61]
Cleithracara maronii		24				[62]
Cleithracara maronii			50			reviewed in [49]
Herichthys cyanoguttatu	Mexico		48			[49]
Herichthys cyanoguttatu	Rioverde (Mexico)		48	6m/sm+42st/a		[61]
Herichthys labridens	Mexico		48	6m/sm+42st/a		[61]
Herichthys minckleyi			48	6m/sm+42st/a		[61]
Heros efasciatus	Araguaia river, Barra do Garças and São Félix do Araguaia (MT, Brazil)		48	4m/sm+44st/a	2	present paper
Heros severus		24				reviewed in [49, 62]
Heros sp.		24				reviewed in [49]
Heros sp.	Marchantaria and Catalão lakes (AM, Brazil)	24				[62]
Herotilapia multispinosa	Commercial source		48			reviewed in [49]
Herotilapia multispinosa			48	6m/sm+42st/a		[61]
Hypselecara coryphaenoides			48	6m/sm+42st/a		[61]
Laetacara curviceps		19				[62]
Laetacara dorsigera	Bahia river, Pracinha (PR, Brazil)		44	4m/sm+40st/a	2	present paper
Mesonauta festivus		24				[62]
Mesonauta festivus			48			reviewed in [49]
Mesonauta festivus			48	8m/sm+40st/a		[61]
Mesonauta festivus	Araguaia river, Barra do Garças and São Félix Araguaia (MT, Brazi)		48	16m/sm+32st/a	6	present paper
Nandopsis tetracanthus	San Juan (Cuba)		48	6m/sm+42st/a		[73]
Nannacara anomala		24				reviewed in [49]
Nannacara anomala		22				[62]
Nannacara anomala			44	18m/sm+26st/a		[61]
Neetroplus nematopus			48	8m/sm+40st/a		[61]
Parachromis dovii	Cuarto river (Costa Rica)		48	8m/sm+40st/a		[61]
Parachromis managuensis			48			reviewed in [49]
Parachromis managuensis			48	6m/sm+42st/a		[61]
Parachromis managuensis	Commercial Source, Brazil		48	6m/sm+42st/a	2	present paper
Pterophyllum scalare		24				reviewed in [49]
Pterophyllum scalare		24				[62]
Pterophyllum scalare	Commercial source		48	4m/sm+44st/a		[61]
Pterophyllum scalare	Jari river, Monte Dourado (PA, Brazil)		48	12m/sm+36st/a	2	[74]
Pterophyllum scalare	Commercial Source, Brazil		48	20m/sm+28st/a	2	present paper
Symphysodon aequifasciatus			60	44m/sm+16st/a		[75]
Symphysodon aequifasciatus		30	60	58m/sm+2st/a		[61, 62]
Symphysodon aequifasciatus	Tefé river; Barcelos (AM, Brazil)		60	48m/sm+8st/a+4 micro	2-3	[31, 44]
Symphysodon aequifasciatus	Commercial Source		60	52m/sm+8st-a		[76]
Symphysodon aequifasciatus	Commercial source, Brazil		60	46m/sm+4st/a+1 0micro		present paper
Symphysodon discus	Negro river, Barcelos (AM, Brazil)		60	50m/sm+10st/a; 54m/sm+6st/a	2-5	[31, 44]
Symphysodon haraldi	Manacapuru river; Barcelos (AM, Brazil)		60	52m/sm+4st/a+4 micro	2-5	[31, 44]
Uaru amphiacanthoides			46	8m/sm+38st/a		[61]

*Presence of supernumerary chromosomes.

**The authors describe the karyotype formula of the species composed of m/sm chromosomes, but the karyotype figure in the paper shows only st/a chromosomes.
